# International trends in arthroscopic hip preservation surgery—are we treating the same patient?

**DOI:** 10.1093/jhps/hnv013

**Published:** 2015-02-18

**Authors:** Brandon J. Erickson, Gregory L. Cvetanovich, Rachel M. Frank, Sanjeev Bhatia, Charles A. Bush-Joseph, Shane J. Nho, Joshua D. Harris

**Affiliations:** 1. Department of Orthopaedic Surgery, Midwest Orthopaedics at Rush, Rush University Medical Center, 1611 West Harrison Street, Suite 300, Chicago, IL 60612, USA; 2. Department of Orthopedics and Sports Medicine, Sports Medicine, Houston Methodist Hospital, 6550 Fannin, Smith Tower, Suite 2500, Houston, TX 77030, USA; 3. Department of Orthopaedic Surgery, Weill Cornell College of Medicine, 1300 York Avenue, New York, NY 10065, USA

## Abstract

The goal of this study was to perform a systematic review and meta-analysis of the entire arthroscopic hip preservation literature to answer the question, ‘Across the world, are we treating the same patient?’ There are significant differences in arthroscopic hip preservation publications, subjects and techniques based on both continent and country published. A systematic review was registered with PROSPERO and performed with PRISMA guidelines using three publicly available databases. Therapeutic clinical outcome investigations reporting arthroscopic hip preservation were eligible for inclusion. All study, subject and surgical technique demographics were analyzed and compared between continents and countries. Statistics were calculated using Student's *t*-tests, one-way analysis of variance, chi-squared and two-proportion *Z*-tests. There were 134 studies included in the analysis (10 752 subjects; 11 007 hips; 51% female; mean 37.6 years of age; mean 27.2 months length of follow-up), which had a low Modified Coleman Methodology Score (mean 32.4; poor). North America published the largest number of studies (58%) and the most subjects (55%) and hips (56%). Australia (22%) and Europe (18%) operated on subjects with some amount of osteoarthritis most commonly. North America (2.7%) and Europe (2.0%) operated on subjects with dysplasia or borderline dysplasia most commonly. The Modified Harris Hip Score was the most frequently utilized outcome score (24% of studies). The quantity and quality of arthroscopic hip preservation literature is significantly increasing with time. Several significant differences in study, subject and surgical technique demographics between continents and countries were identified. Deficiencies in use of clinical outcome scores and definitions of treated pathologies preclude complete subject comparisons and serve as an impetus for future study quality improvements.

## INTRODUCTION

Hip arthroscopy may be used to address a multitude of intra-articular hip pathologies. Although indications for both primary and revision arthroscopy continue to evolve, the most common indication remains treatment of symptomatic femoroacetabular impingement (FAI) and labral injury [3–5]. The number of arthroscopic hip procedures is significantly increasing across the world [6–8]. However, patient demographics, surgical techniques and clinical outcomes utilized are geographically unique [9]. Further, study design, conduct and reporting are also variable.

The purpose of this investigation was to perform a systematic review and meta-analysis of the entire arthroscopic hip preservation literature to identify and compare the summative characteristics of the studies published, subjects analyzed and surgical techniques performed across continents and countries. In essence, the purpose of the study was to answer the question, ‘Across the world, are we treating the same patient?’ The authors hypothesized that there are significant differences in arthroscopic hip preservation publications, subjects and techniques based on both continent and country published.

## METHODS

A systematic review was conducted according to PRISMA guidelines (Preferred Reporting Items for Systematic reviews and Meta-Analyses) using a PRISMA checklist [10]. Systematic review registration was performed using the PROSPERO International prospective register of systematic reviews [11]. Two reviewers independently conducted the search on 28 December 2013 using the following databases: Medline, Cochrane Central Register of Controlled Trials, SportDiscus and CINAHL. The electronic search citation algorithm utilized was ((((((((((((hip[Title/Abstract]) OR labral[Title/Abstract])) OR ((femoroacetabular[Title/Abstract]) AND arthroscopy[Title/Abstract]))) NOT shoulder[Title/Abstract]) NOT subacromial[Title/Abstract]) NOT elbow[Title/Abstract]) NOT wrist[Title/Abstract]) NOT hand[Title/Abstract]) NOT knee[Title/Abstract]) NOT ankle[Title/Abstract]) NOT foot[Title/Abstract] AND (English[lang]). English language Level I–IV evidence 2011 update by the Oxford Centre for Evidence-Based Medicine [12]) clinical studies were eligible. Length of clinical follow-up was not an exclusion criterion. Medical conference abstracts were ineligible for inclusion. All references within included studies were cross-referenced for inclusion if missed by the initial search. Duplicate subject publications within separate unique studies were not reported twice. The study with longer duration follow-up, higher level of evidence or greater number of subjects was retained for inclusion. Level V evidence reviews, letters to the editor, basic science, biomechanical studies, open hip surgery, imaging, surgical technique and classification studies were excluded. The senior author resolved conflicts by reviewers selecting papers.

Participants/subjects/patients of interest in this systematic review underwent hip arthroscopy for intra- or extra-articular pathology (labral tear, FAI, arthritis, hip dysplasia, intra-articular loose body, chondral defect, femoral head fracture, among others). Study and subject demographic parameters analyzed included year of publication, years of subject enrollment, presence of study financial conflict of interest, number of subjects and hips, gender, age, body mass index (BMI), diagnoses treated and surgical positioning. Clinical outcome scores sought were the non-arthritic hip score (NAHS), iHOT-12 or 33 (international Hip Outcome Tool - 12 or 33), Hip Outcome Score (HOS - activities of daily living and Sports), modified Harris Hip Score (MHHS), Hip disability and Osteoarthritis Outcome Score, SF-12 (Short-Form), University of California Los Angeles (UCLA) activity score, Tegner activity score and Merle d'Aubigne Postel score. Plain radiographic, computed tomography and magnetic resonance imaging (MRI) data were extracted when available. As with several other systematic reviews, study methodological quality was evaluated using the Modified Coleman Methodology Score (MCMS) [9, 13–15].

### STATISTICAL ANALYSIS

Study descriptive statistics were calculated. Continuous variable data were reported as mean ± standard deviation from the mean. Weighted means and standard deviations were calculated for all subject, hip and surgical parameters. Categorical variable data were reported as frequency with percentages. For all statistical analysis either measured and calculated from study data extraction or directly reported from the individual studies, *P* < 0.05 was considered statistically significant. For continuous data across continents and countries, one-way analysis of variance was utilized to compare groups. For categorical data across continents and countries, chi-square analysis was utilized to compare groups. Where applicable, study, subject and surgical outcomes data were also compared using two-sample and two-proportion *Z*-test calculators with alpha 0.05 because of the difference in sample sizes between compared groups.

## RESULTS

**Fig. 1.**
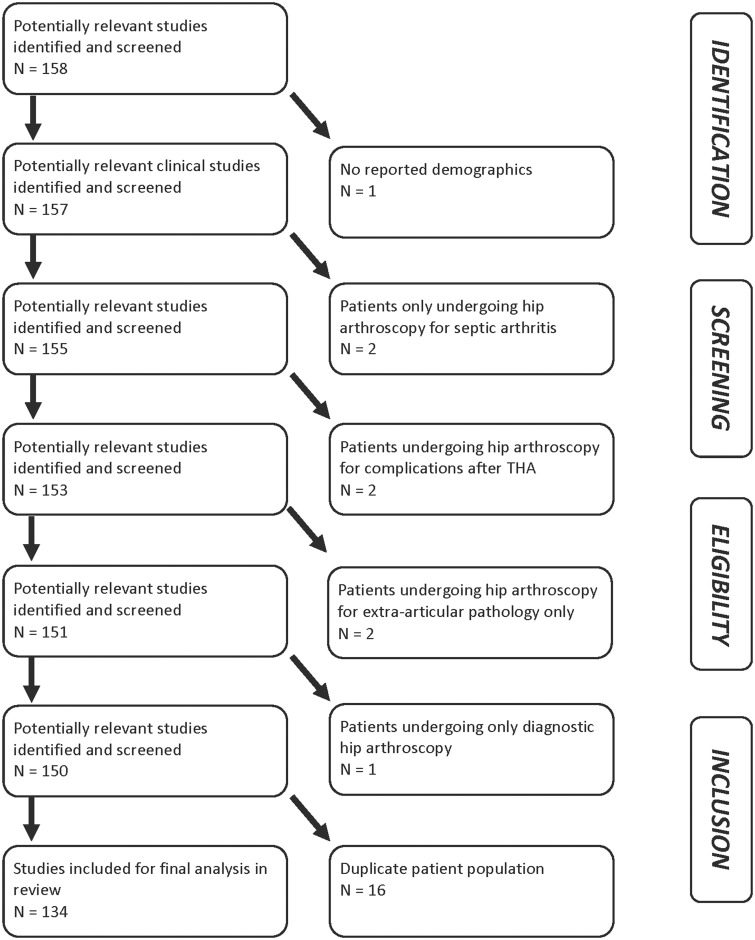
PRISMA flowchart.

**Fig. 2.**
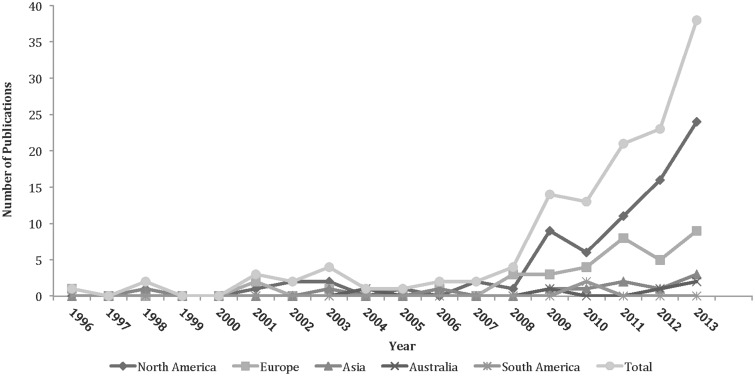
Number of publications per continent over time.

**Fig. 3.**
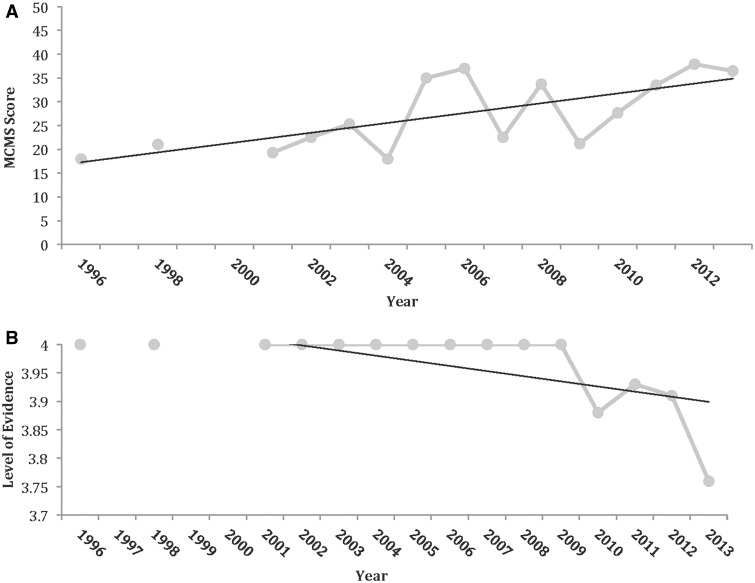
(A) Mean MCMS over time for all continents combined. (B) Mean level of evidence over time for all continents combined. Note that lower numerical level of evidence is observed over later publication date, indicative of improved, rather than worse, level of evidence.

**Fig. 4.**
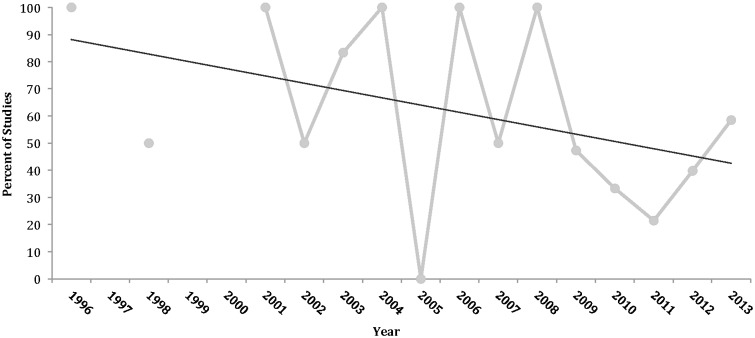
Present or not reported financial conflict of interest over time for all continents combined. COI, conflict of interest.

In total, 134 studies were included in the analysis (Fig. 1) (10 752 subjects; 11 007 hips; 51% female; mean 37.6 years of age; mean 27.2 months length of follow-up) (See Supplementary data). Most studies were Level IV evidence (88%), had a low MCMS (mean 32.4; poor) and were single-center investigations (93%) without a declared financial conflict of interest (52%) (Table I). The percentage of males and females reported in studies from varying continents was not significantly different. From 1996 to 2013, among all continents, the number of publications significantly increased with time (Fig. 2), the MCMS significantly increased (Fig. 3A) and the level of evidence significantly improved (Fig. 3B). Although there were fewer publications with financial conflicts of interest reported over time, the temporal trend was not significant (Fig. 4).

**Table I.** Demographic data by continent

	North America	South America	Europe	Asia	Australia
Number of studies	78	3	37	11	5
Level of evidence	3.78 ± 0.62	3.33 ± 1.15	3.89 ± 0.39	4 ± 0	3.8 ± 0.45
I	2	0	0	0	0
II	2	1	1	0	0
II	7	0	2	0	1
IV	67	2	34	11	4
MCMS	32.7 ± 10.9	36.7 ± 9.3	33.0 ± 11.8	29.6 ± 10.9	30 ± 11.8
Financial conflict of interest					
Present	25	1	5	0	1
Not present	39	2	22	7	0
Not reported	14	0	10	4	4
Institutional collaboration					
Single center	70	3	35	11	5
Multi-center	8	0	2	0	0
Number of subjects	5912	367	3607	195	671
Male^a^	2679 (49.6%)	187 (51.0%)	1601 (46.6%)	97 (49.7%)	341 (50.8%)
Female	2724 (50.4%)	180 (49.0%)	1832 (53.4%)	98 (50.3%)	330 (49.2%)
Number of hips	6124	367	3641	197	678
Mean age (years)	34.4 ± 11.1	34.7 ± 1.70	37.2 ± 13.1	41 ± 13.4	40.5 ± 17.9
Minimum age (mean across studies)	23.5 ± 12.9	14.7 ± 20.1	20.8 ± 12.9	27.7 ± 15.6	21.3 ± 17.8
Maximum age (mean across studies)	46.3 ± 18.3	61 ± 16.5	53.6 ± 19.8	52.1 ± 17.5	41 ± 33.9
Mean BMI (kg/m^2^)	26.9 (17 studies/2289 subjects)	23.7 (1 study/166 subjects)	26.8 (5 studies/168 subjects)	23.2 (1 study/40 subjects)	nr
Mean length of follow-up (months)	27.4 ± 24.4	31.8 ± 11.0	25.3 ± 22.6	30.5 ± 34.7	21.0 ± 21.6
Number of studies reporting pre-operative CT scan (number of subjects)	17 (382)	0	4 (146)	4 (38)	2 (104)
Number of studies reporting pre-operative MRI scan (number of subjects)	47 (3625)	1 (7)	22 (1191)	6 (123)	3 (105)

CT, computed tomography; nr, not recorded.

^a^Gender not reported in all subjects across studies.

Among continents, North America published the largest number of studies (58%), along with the largest number of subjects (55%) and hips (56%) (Table I). Among countries, the United States published the largest number of studies (54%), along with the largest number of subjects (52%) and hips (53%) (Table II). The next four most published countries were England, Switzerland, Germany and Australia.

**Table II.** Demographic data by country

Country	USA	UK	Switzerland	Germany	Australia
Number of studies	72	11	7	7	5
Number of subjects (hips)	5637 (5830)	2396 (2412)	307 (318)	115 (118)	671 (678)
Level of evidence	3.76 ± 0.64	4	3.86 ± 0.38	4	3.8 ± 0.45
I	2	0	0	0	0
II	2	0	0	0	0
III	7	0	1	0	1
IV	61	11	6	0	4
MCMS	32.7 ± 11.0	39.7 ± 7.6	32.7 ± 13.0	24.3 ± 13.2	30 ± 11.8
Financial conflict of interest					
Present	23	3	1	0	1
Not present	36	7	3	3	0
Not reported	13	1	3	4	4
Institutional Collaboration					
Single center	65	11	7	7	5
Multi-center	7	0	0	0	0
Number of subjects					
Male^a^	2552	1065	57	36	341
Female	2561	1336	109	44	330
Mean age (years)	34.2 ± 10.8	36.7 ± 11.1	34.5 ± 11.4	36.2 ± 19.0	40.5 ± 17.9
Minimum age (mean across studies)	23.0 ± 12.6	21.7 ± 15.1	17.3 ± 4.0	23.2 ± 16.0	21.3 ± 17.8
Maximum age (mean across studies)	45.3 ± 18.4	64.6 ± 11.1	50.2 ± 19.7	42 ± 18.1	50.3 ± 34.7
Mean BMI (kg/m^2^)	26.9	28.2	24.5	Nr	Nr
Mean length of follow-up (months)	27.8 ± 25.3	28.0 ± 20.4	16.3 ± 13.4	20.6 ± 11.0	21.0 ± 21.6

Nr, not recorded.

^a^when gender was specified in articles.

The most common surgical indication was painful FAI with or without labral injury. Cam (Table III) and pincer impingement (Table IV), labral tears, hip dysplasia (Table V) and arthritis (Table III) were very poorly defined across all continents and countries. Cam and pincer impingement, dysplasia and arthritis were poorly defined across all locations (defined in 17%, 19%, 16% and 59% of studies, respectively). Of all subjects undergoing hip arthroscopy within a certain geographic location, the proportion of subjects reported that underwent surgery with varying amounts of osteoarthritis osteoarthritis was greatest in Australia (22%) and Europe (18%). Of all subjects undergoing hip arthroscopy within a certain geographic location, the proportion of subjects reported that underwent surgery for dysplasia or borderline dysplasia was greatest in North America (2.7%) and Europe (2.0%) (Table VI). North America, South America and Asia performed hip arthroscopy most frequently in the supine position, whereas Europe and Australia used predominantly the lateral position.

**Table III:** Definition of CAM impingement and arthritis across continents

	North America	South America	Europe	Asia	Australia
*Definition of cam impingement*					
Number of studies reporting (subjects) (% of studies reporting a definition)	14 (949) (18%)	0	7 (337) (9%)	1 (21) (9%)	1 (34) (20%)
Alpha angle > 45° (oblique)	1 (36)		0	0	0
Alpha angle > 50° (Dunn 90°)	3 (67)		0	0	0
Alpha angle > 50° (Cross-table lateral)	1 (60)		0	0	0
Alpha angle > 50° (Cross-table lateral, frog-leg lateral)	0		0	1 (21)	0
Alpha angle > 50° (Cross-table lateral, Dunn 45°)	0		1 (110)	0	0
Alpha angle > 50° (AP, cross-table lateral)	1 (185)		0	0	0
Alpha angle > 50° (AP, cross-table lateral, Dunn 45°)	1 (210)		0	0	0
Alpha angle > 55° (MRI, axial oblique)	0		2 (42)	0	0
Alpha angle > 55°	1 (9)		0	0	0
Alpha angle > 55° (cross-table lateral)	2 (126)		1 (96)	0	0
Alpha angle > 55° (MRI, axial oblique)	1 (50)		0	0	0
Alpha angle > 55° (MRI, radial)	0		1 (38)	0	0
Alpha angle > 55° (Cross-table lateral, or CT scan)	0		0	0	1 (34)
Alpha angle > 60° (AP, frog-leg lateral, or Dunn 90°)	1 (58)		0	0	0
Qualitative (Femoral head asphericity)	2 (148)		1 (40)	0	0
Qualitative (Pistol-grip deformity)	0		1 (11)	0	0
Mean alpha angle (degrees)	63.8 ± 8.70	nr	67.6 ± 9.39	65.7 ± 8.5	nr
Number of studies reported (subjects)	13 (2666)		5 (256)	1 (21)	
Proportion of studies reporting (subjects)	17% (45%)		14% (7.1%)	9.1% (11%)	
*Definition of arthritis*					
Number of studies defining hip arthritis (number of hips)	46 (3099) (59%)	0	24 (2203) (65%)	7 (180) (64%)	2 (630) (40%)
Via Tonnis grade	16 (1632)		12 (435)	4 (66)	0
≥2	15 (1526)		12 (435)	4 (66)	
≥1	1 (106)		0	0	0
Via joint space narrowing	5 (345)		0	1 (21)	
<3 mm	2 (172)		0	0	
<2 mm	2 (172)		0	0	1 (560)
‘Joint space narrowing’	1 (1)		0	1 (21)	0
Via Outerbridge classification	3 (291)		1 (94)	0	1 (70)
Via ‘arthritis’ or ‘osteoarthritis’	18 (776)		9 (1513)	2 (93)	0
Via ‘degenerative changes’	3 (19)		0	0	0
Via ‘cartilage delamination’	1 (36)		0	0	
Via Kellgren–Lawrence grade	0		1 (11)	0	
Number of hips with arthritis (%)	312 (10.1%)	nr	387 (17.6%)	16 (8.9%)	137 (21.7%)

CT, computed tomography; nr, not recorded.

**Table IV.** Definition of pincer impingement across continents

	North America	South America	Europe	Asia	Australia
*Definition of pincer impingement*					
Number of studies reporting (subjects) (% of studies reporting a definition)	16 (949) (21%)	0	8 (471) (22%)	1 (21) (9%)	0
Crossover sign	9 (535)		7 (353)	1 (21)	
Coxa profunda	7 (522)		4 (182)	1 (21)	
Protrusio acetabulae	7 (430)		4 (182)	0	
Acetabular retroversion	3 (305)		1 (118)^a^	1 (21)	
LCEA > 40°	2 (150)		0	1 (21)	
ACEA > 35°	1 (60)		0	0	
Tönnis angle < 0°	1 (60)		0	0	
Ischial spine sign	1 (36)		1 (101)	0	
Posterior wall sign	0		1 (11)	0	
Mean lateral center edge angle (degrees)	31.2 ± 10.8	nr	31.7 ± 6.71	nr	nr
Number of studies reported (subjects)	12 (1747)		3 (215)		
Proportion of studies reporting (subjects)	15% (30%)		8.1% (6.0%)		
Mean anterior center edge angle (degrees)	28.5 ± 3.54	nr	nr	nr	nr
Number of studies reported (subjects)	2 (110)				
Proportion of studies reporting (subjects)	2.6% (1.9%)				
Mean Tönnis angle	7.0°	nr	12.8°	nr	nr
Number of studies reported (subjects)	4 (26)		1 (86)		
Proportion of studies reporting (subjects)	5.1% (0.4%)		2.7% (2.4%)		
Number of studies assessing AIIS impingement (subspine impingement)	4 (228)	nr	nr	nr	nr

ACEA, anterior center edge angle; AIIS, anterior inferior iliac spine; LCEA, lateral center edge angle; nr, not recorded.

^a^Retroversion defined as anteversion less than 14 degrees.

**Table V.** Definition of hip dysplasia across continents

	North America	South America	Europe	Asia	Australia
*Definition of hip dysplasia*					
Number of studies defining hip dysplasia (number of hips)	16 (1,048) (21%)	0	5 (473) (14%)	0	0
Via LCEA < 25°	4 (176)		3 (276)		
Via LCEA < 20°	7 (457)		1 (111)		
Via ACEA < 20°	2 (61)				
Via Tönnis angle > 10°	2 (110)				
Via acetabular index < 20°	1 (36)				
Via “dysplasia”	2 (251)				
Via femoral head coverage (CT/MRI)	1 (1)				
Via hypertrophic labrum (MR, arthroscopy)	1 (1)				
Via anterior or posterior femoral head surface area <10%	1 (36)		1 (86)		
Via Type I (incongruent) or II (short arc)	13 (320)		8 (366)	1 (7)	1 (1)
Not defined, but reported the number of subjects with dysplasia					
Number of studies defining borderline hip dysplasia (number of hips)		0		0	0
Via LCEA 20°–25°	4 (252)		1 (111)		
Via LCEA 18°–25°	1 (22)		0		
Via ACEA 20°–25°	1 (106)		0		
Via Sharp’s angle > 40°	1 (106)		0		
Number of subjects with hip dysplasia	82	nr	63	7	0
Number of subjects with borderline hip dysplasia	78	nr	10	0	1
Number of studies that excluded dysplastic hips from investigation (proportion)	10 (13%)	nr	5 (14%)	0	1 (20%)

ACEA, anterior center edge angle; LCEA, lateral center edge angle; CT, computed tomography; nr, not recorded.

**Table VI.** Indications for surgery across continents

Indications for surgery, number of studies (number of subjects)	North America	South America	Europe	Asia	Australia
FAI	26 (1971)	1 (194)	8 (712)	1 (73)	
Labral tear	18 (1912)	1 (194)	2 (51)	2 (22)	1 (70)
Arthritis	1 (60)		1 (22)		
Dysplasia	2 (25)				
Coxa saltans	3 (59)	1 (7)	1 (15)		
Pain	19 (2190)	1 (166)	18 (2654)	1 (40)	3 (602)
Trauma (femoral head fracture)	1 (2)			1 (11)	
Trauma (hip dislocation)	3 (40)		1 (8)		
Loose bodies	5 (5)		1 (4)	3 (22)	
Revision hip arthroscopy	1 (60)				
Ligamentum teres tear	1 (4)				
Pigmented villonodular synovitis	1 (13)		1 (1)		
Synovial chondromatosis			2 (122)	1 (24)	
Abductor tendon tear	1 (11)				
Septic arthritis					1 (6)
Symptomatic paralabral cyst				1 (2)	
Calcified rectus femoris tendon				1 (3)	
Chondral defects			2 (55)		

The use of clinical outcome scores was poor across all locations. Fifty-nine (44%) studies utilized one or more score(s) in reporting their clinical outcomes. Although the MHHS was the most frequently utilized outcome score, it was only used in 32 (24%) studies. The NAHS (7–89%) and HOS (0.1–7.6%), among others, were less frequently reported. There was a significant difference in MHHS between continents (*P* = 0.01) (Table VII). There was no significant difference between continents in reference to study MCMS (*P* = 0.83), study length of follow-up (0.96), study level of evidence (*P* = 0.29), subject gender (*P* = 0.69), subject age (*P* = 0.52), subject BMI (*P* = 0.65), subject mean alpha angle (*P* = 0.73) and subject mean lateral center edge angle (*P* = 0.94).

**Table VII.** Most utilized outcome scores across continents

Outcome score	North America	South America	Europe	Asia	Australia
NAHS	42.0 ± 17.8 (6; 440; 7.2%)		48.9 ± 4.96 (9; 422; 11.6%)		67.1 ± 7.0 (2; 601; 89%)
HOS					
Activities of daily living	70.0 ± 11.7 (8; 398; 6.5%)		52.8 (1; 4; 0.1%)		
Sports	44.1 ± 6.34 (9; 463; 7.6%)		nr		
Modified HHS	61.6 ± 6.51 (23; 2519; 41%)	56.1 (1; 7; 1.9%)	58.2 ± 13.7 (7; 1006; 28%)	46.1 (1; 40; 20%)	68.0 ± 7.1 (2; 601; 89%)
HHS				50.4 ± 13.2 (3; 47; 24%)	
Hip disability and Osteoarthritis Outcome Score					
Pain			46.4 (1; 94; 2.6%)		
Symptoms			44.2 (1; 94; 2.6%)		
Activities of daily living			51.1 (1; 94; 2.6%)		
Sports and recreation			30.7 (1; 94; 2.6%)		
Quality of life			39.6 (1; 94; 2.6%)		
WOMAC	36.6 ± 7.8 (3; 180; 2.9%)		47.2 ± 20.4 (3; 172; 4.7%	65 (1; 2; 1.0%)	
Tegner activity score			7.6 (1; 15; 0.4%)		
SF-12					
PCS	46.6 ± 18.0 (3; 1503; 25%)				
MCS	53 (1; 1264; 21%)				
iHOT-12					
iHOT-33					
SF-36					
EQ-5-QoL					
Marx activity score					

nr, not recorded.

Blank cells indicate that no clinical outcome score was utilized. In parentheses: (number of studies reporting this variable; number of subjects with this variable measured; proportion of subjects within overall geographic area with that variable measured).

## DISCUSSION

Hip arthroscopy is an emerging surgical technique used to treat a multitude of hip pathologies across the world. The authors sought to identify and compare hip arthroscopy studies, subjects and surgeries across all countries and continents. In essence, the study's primary purpose was to answer the question, ‘Are we all treating the same patient?’ The study hypotheses were confirmed in that several significant differences in study, subject and surgical technique demographics between continents and countries were identified, whereas primary question was left unanswered. Unfortunately, deficiencies in use of clinical outcome scores and definitions of treated pathologies (impingement, arthritis, dysplasia) preclude complete subject comparisons and serve as an impetus for future study improvements.

This study demonstrated that the mean level of evidence and quality of studies surrounding hip arthroscopy is poor per the MCMS and 88% of studies were Level IV evidence. When most of the literature about a specific procedure is made up of low evidence work, it is difficult to draw concrete conclusions about the results of this procedure. With the evolving landscape of medial reimbursements, orthopedic sports medicine specialists who perform hip arthroscopy will need to design higher level studies to validate the outcomes of this procedure. The future of hip surgery may center around proving that hip arthroscopy is beneficial based on a validated outcome score. Furthermore, a standardized approach to defining the pathology addressed by hip arthroscopy is necessary to ensure patients with adequate pathology are indicated for this procedure, thereby attempting to ensure significant clinical improvement after this procedure and enabling comparison of results across nations.

When studies from across the world are reporting outcomes for hip arthroscopy patients, they may be reporting these outcomes on dissimilar patients, thereby skewing the results from one region compared with another. Studies have reported on large-scale series in individual countries as it relates to hip arthroscopy [16]. Clohisy *et al. [*8] had a case series of 1130 hips who underwent surgical intervention for FAI and found that the majority of patients were white, with a slight female predominance (55% vs. 45%). In that study, FAI was defined by each individual surgeon but was broadly classified by ‘abnormal repetitive abutment of the proximal femur and acetabular rim that led to patient- reported dysfunction of the hip’. There are numerous studies like this that do not quantify the measurements used to define FAI and therefore lead to a wide variability in the patients who are treated with hip arthroscopy [17]. Varying the indications could have an effect on the reported outcomes.

One of the salient outcomes this study identified was the lack of consensus on defining cam and pincer morphology, arthritis and dysplasia. Although Australia and North America reported this definition more then Asia, Europe and South America, there was no consistency with this reporting based on alpha angle, or the X-ray or MRI view used to determine the alpha angle (Tables III–V). Although no consistent definition of a cam deformity exists in the literature, many authors use an alpha angle of >60° to define this lesion. However, the method of measuring this alpha angle is subject to variability, as some authors use MRI as originally described, whereas others use anteroposterior, Dunn or crosstable radiographs. Studies have shown that the Dunn view most closely approximates MRI but that one can still miss anterior or posterior-based cam lesions depending on the views used [18].

Acetabular deformity and handling of the labrum are important pathologies necessitating treatment during hip arthroscopy. Larson *et al. *[19, 20] reported on the short- and mid-term results of patients who underwent hip arthroscopy for pincer-type impingement and found that patients who underwent labral refixation had significantly higher outcomes scores than patients who underwent labral excision/debridement. This is important for surgeons to recognize as labral lesions should be fixed in patients with pincer-type impingement when possible. Similarly, in performing a hip arthroscopy on patients with hip dysplasia, debridement of the labrum instead of refixation has been associated with higher failure rate than labral repair [1, 21]. This study found more literature that reported preoperative diagnoses of osteoarthritis (852 patients) than dysplasia (150 patients) in patients undergoing hip arthroscopy, although both are risk factors for failure from hip arthroscopy.

Interestingly, the definition of arthritis was given in a majority of studies, although the definition was not consistent throughout the various continents (Table III). As hip arthritis is a well-documented cause of failure from hip arthroscopy, it is interesting that approximately 20% of patients with evidence of arthritis underwent hip arthroscopy in Europe and Australia, whereas other continents reported much lower percentages [22, 23]. Surgeons may need to be educated on this fact to avoid failures in patients with arthritis. Skendzel *et al. *[2] recently reported on the conversion rates of post-operative hip arthroscopy patients to total hip arthroplasty (THA) and found that, in a series of 466 patients, 86% of patient with limited joint space (defined as <2 mm of joint space on anteroposterior pelvis radiographs) had undergone THA, whereas only 16% of patients with preserved joint space (>2 mm of joint space) underwent THA. Shearer *et al. *[24] found that hip arthroscopy was cost effective as it related to quality of life if it delayed progression to a THA for more than 16 years after the arthroscopic procedure.

There are numerous outcome scores that are used to characterize pain and function about the hip. There was a significant amount of variability in the outcome scores that were used across the world, with many areas not using any validated outcome scores at all (Table VII). In fact, 56% of studies did not use any clinical outcome score at all in reporting their results, whereas the MHHS score was used most frequently (24% of studies). Interestingly, Tijssen *et al. *[25] performed a systematic review that examined studies using the MHHS, HOS and NAHS to determine which patient reported outcome questionnaire was valid and reliable in the evaluation of patients undergoing hip arthroscopy and found the NAHS was the best quality questionnaire for these patients. They recommended surgeons use a combination of the HOS and NAHS for evaluating patients undergoing hip arthroscopy. This study found the HOS was used sparingly in North America and Europe, whereas the NAHS was used more frequently in North America, Europe and Australia. However, the MHHS was the most consistently used outcome score across all nations. As the MHHS has been in existence longer than the NAHS and HOS, many surgeons may be more familiar with the MHHS score and so are more apt to use this score than others [26].

### LIMITATIONS

Although this study reviewed all literature pertinent to hip arthroscopy, there are limitations. Some studies could have been missed, despite the fact that two authors performed the search. Agreement statistics were not performed between reviewers. There were many studies that did not report on all the variables the authors examined. This study did not address outcome measures and so cannot draw conclusions on the best treatment options, surgical positioning, etc. This study also did not examine any concomitant pathology at the time of surgery as the primary aim was not to analyze outcomes.

## CONCLUSION

The quantity and quality of arthroscopic hip preservation literature is significantly increasing with time. Several significant differences in study, subject and surgical technique demographics between continents and countries were identified. Many geographic similarities were identified in subject demographics. However, deficiencies in use of clinical outcome scores and definitions of treated pathologies (impingement, arthritis, dysplasia) preclude complete subject comparisons and serve as an impetus for future study quality improvements.

## SUPPLEMENTARY DATA

Supplementary data are available at *Journal of Hip Preservation Surgery* online.

## CONFLICT OF INTEREST STATEMENT

Brandon J Erickson Gregory L Cvetanovich, Rachel M Frank, and Sanjeev Bhatia have no conflicts of interest.

## Supplementary Material

Supplementary Data

## References

[1] Parvizi J Bican O Bender B Arthroscopy for labral tears in patients with developmental dysplasia of the hip: a cautionary note. J Arthroplasty 2009; 24(Suppl. 6): 110–3. 19596542 10.1016/j.arth.2009.05.021

[2] Skendzel JG Philippon MJ Briggs KK The effect of joint space on midterm outcomes after arthroscopic hip surgery for femoroacetabular impingement. Am J Sports Med 2014; 42: 1127–33. 24607652 10.1177/0363546514526357

[3] Philippon MJ Ferro FP Nepple JJ Hip capsulolabral spacer placement for the treatment of severe capsulolabral adhesions after hip arthroscopy. Arthrosc Tech 2014; 3: e289–92. 24904779 10.1016/j.eats.2014.01.003 PMC4044510

[4] Sansone M Ahlden M Jonasson P A Swedish hip arthroscopy registry: demographics and development. Knee Surg Sports Traumatol Arthrosc 2014; 22: 774–80. 24464406 10.1007/s00167-014-2840-9

[5] Reich MS Shannon C Tsai E Hip arthroscopy for extra-articular hip disease. Curr Rev Musculoskelet Med 2013; 6: 250–7. 23881610 10.1007/s12178-013-9177-8 PMC4094012

[6] Bozic KJ Chan V Valone FH 3rd Trends in hip arthroscopy utilization in the United States. J Arthroplasty 2013; 28(Suppl. 8): 140–3. 23916639 10.1016/j.arth.2013.02.039

[7] Lee YK Ha YC Yoon BH National trends of hip arthroscopy in Korea. J Korean Med Sci 2014; 29: 277–80. 24550658 10.3346/jkms.2014.29.2.277 PMC3924010

[8] Clohisy JC Baca G Beaule PE Descriptive epidemiology of femoroacetabular impingement: a North American cohort of patients undergoing surgery. Am J Sports Med 2013; 41: 1348–56. 23669751 10.1177/0363546513488861

[9] Harris JD McCormick FM Abrams GD Complications and reoperations during and after hip arthroscopy: a systematic review of 92 studies and more than 6,000 patients. Arthroscopy 2013; 29: 589–95. 23544691 10.1016/j.arthro.2012.11.003

[10] Liberati A Altman DG Tetzlaff J The PRISMA statement for reporting systematic reviews and meta-analyses of studies that evaluate health care interventions: explanation and elaboration. J Clin Epidemiol 2009; 62: e1–34. 19631507 10.1016/j.jclinepi.2009.06.006

[11] The University of York CfRaDP-Iprosr-v. 2013 [cited 2014]. Available at: http://www.crd.york.ac.uk/PROSPERO/, accessed May 5, 2014.

[12] The University of Oxford CfEBMOLoES. Oxford Centre for Evidence Based Medicine, 2012.

[13] Cowan J Lozano-Calderon S Ring D Quality of prospective controlled randomized trials. Analysis of trials of treatment for lateral epicondylitis as an example. J Bone Joint Surg Am 2007; 89: 1693–9. 17671006 10.2106/JBJS.F.00858

[14] Gupta AK Harris JD Erickson BJ Surgical management of complex proximal humerus fractures—a systematic review of 92 studies including 4,500 patients. J Orthop Trauma 2015; 29: 54–9. 25162974 10.1097/BOT.0000000000000229

[15] Harris JD Erickson BJ Bush-Joseph CA Treatment of femoroacetabular impingement: a systematic review. Curr Rev Musculoskelet Med 2013; 6: 207–18. 23743861 10.1007/s12178-013-9172-0 PMC4094011

[16] Willimon SC Briggs KK Philippon MJ Intra-articular adhesions following hip arthroscopy: a risk factor analysis. Knee Surg Sports Traumatol Arthrosc 2014; 22: 822–5. 24162717 10.1007/s00167-013-2728-0

[17] Clarke MT Arora A Villar RN Hip arthroscopy: complications in 1054 cases. Clin Orthop Relat Res 2003; 406: 84–8. 12579004 10.1097/01.blo.0000043048.84315.af

[18] Barton C Salineros MJ Rakhra KS Validity of the alpha angle measurement on plain radiographs in the evaluation of cam-type femoroacetabular impingement. Clin Orthop Relat Res 2011; 469: 464–9. 20953854 10.1007/s11999-010-1624-x PMC3018186

[19] Larson CM Giveans MR Arthroscopic debridement versus refixation of the acetabular labrum associated with femoroacetabular impingement. Arthroscopy 2009; 25: 369–76. 19341923 10.1016/j.arthro.2008.12.014

[20] Larson CM Giveans MR Stone RM Arthroscopic debridement versus refixation of the acetabular labrum associated with femoroacetabular impingement: mean 3.5-year follow-up. Am J Sports Med 2012; 40: 1015–21. 22307078 10.1177/0363546511434578

[21] Philippon MJ Schenker ML Briggs KK Revision hip arthroscopy. Am J Sports Med 2007; 35: 1918–21. 17703000 10.1177/0363546507305097

[22] Bogunovic L Gottlieb M Pashos G Why do hip arthroscopy procedures fail? Clin Orthop Relat Res 2013; 471: 2523–9. 23637056 10.1007/s11999-013-3015-6 PMC3705062

[23] Philippon MJ Briggs KK Carlisle JC Joint space predicts THA after hip arthroscopy in patients 50 years and older. Clin Orthop Relat Res 2013; 471: 2492–6. 23292888 10.1007/s11999-012-2779-4 PMC3705033

[24] Shearer DW Kramer J Bozic KJ Is hip arthroscopy cost-effective for femoroacetabular impingement? Clin Orthop Relat Res 2012; 470: 1079–89. 21842295 10.1007/s11999-011-2023-7 PMC3293959

[25] Tijssen M van Cingel R van Melick N Patient-Reported Outcome questionnaires for hip arthroscopy: a systematic review of the psychometric evidence. BMC Musculoskelet Disord 2011; 12: 117. 21619610 10.1186/1471-2474-12-117 PMC3129322

[26] Harris WH Etiology of osteoarthritis of the hip. Clin Orthop Relat Res 1986; 213: 20–33. 3780093

