# Supplementary material for: International trends in arthroscopic hip preservation surgery—are we treating the same patient?
Source: J Hip Preserv Surg. 2015 Feb 18;2(1):28–41. doi: 10.1093/jhps/hnv013 (PMC4718469; doi:10.1093/jhps/hnv013)
Supplement: Supplementary Data [file supp_2_1_28__index.html]

Supplementary Data 

# International trends in arthroscopic hip preservation surgery—are we treating the same patient?

## Supplementary Data

files

**Files in this Data Supplement:**

- Supplementary Data - docx file
