# Supplementary material for: International trends in arthroscopic hip preservation surgery—are we treating the same patient?
Source: J Hip Preserv Surg. 2015 Feb 18;2(1):28–41. doi: 10.1093/jhps/hnv013 (PMC4718469; doi:10.1093/jhps/hnv013)
Supplement: Supplementary Data [file supp_hnv013_Appendix_1.docx]

(1-134)

1. Krych AJ, Thompson M, Knutson Z, Scoon J, Coleman SH. Arthroscopic labral repair versus selective labral debridement in female patients with femoroacetabular impingement: a prospective randomized study. Arthroscopy : the journal of arthroscopic & related surgery : official publication of the Arthroscopy Association of North America and the International Arthroscopy Association. 2013;29(1):46-53.

2. Mei-Dan O, McConkey MO, Knudsen JS, Brick MJ. Bilateral hip arthroscopy under the same anesthetic for patients with symptomatic bilateral femoroacetabular impingement: 1-year outcomes. Arthroscopy : the journal of arthroscopic & related surgery : official publication of the Arthroscopy Association of North America and the International Arthroscopy Association. 2014;30(1):47-54.

3. Byrd JW, Jones KS, Maiers GP, 2nd. Two to 10 Years' follow-up of arthroscopic management of pigmented villonodular synovitis in the hip: a case series. Arthroscopy : the journal of arthroscopic & related surgery : official publication of the Arthroscopy Association of North America and the International Arthroscopy Association. 2013;29(11):1783-7.

4. Domb BG, Stake CE, Botser IB, Jackson TJ. Surgical dislocation of the hip versus arthroscopic treatment of femoroacetabular impingement: a prospective matched-pair study with average 2-year follow-up. Arthroscopy : the journal of arthroscopic & related surgery : official publication of the Arthroscopy Association of North America and the International Arthroscopy Association. 2013;29(9):1506-13.

5. Domb BG, Stake CE, Lindner D, El-Bitar Y, Jackson TJ. Arthroscopic capsular plication and labral preservation in borderline hip dysplasia: two-year clinical outcomes of a surgical approach to a challenging problem. The American journal of sports medicine. 2013;41(11):2591-8.

6. Telleria JJ, Safran MR, Harris AH, Gardi JN, Glick JM. Risk of sciatic nerve traction injury during hip arthroscopy-is it the amount or duration? An intraoperative nerve monitoring study. The Journal of bone and joint surgery American volume. 2012;94(22):2025-32.

7. Safran MR, Epstein NP. Arthroscopic management of protrusio acetabuli. Arthroscopy : the journal of arthroscopic & related surgery : official publication of the Arthroscopy Association of North America and the International Arthroscopy Association. 2013;29(11):1777-82.

8. Palmer DH, Ganesh V, Comfort T, Tatman P. Midterm outcomes in patients with cam femoroacetabular impingement treated arthroscopically. Arthroscopy : the journal of arthroscopic & related surgery : official publication of the Arthroscopy Association of North America and the International Arthroscopy Association. 2012;28(11):1671-81.

9. Willimon SC, Briggs KK, Philippon MJ. Intra-articular adhesions following hip arthroscopy: a risk factor analysis. Knee surgery, sports traumatology, arthroscopy : official journal of the ESSKA. 2014;22(4):822-5.

10. Hapa O, Bedi A, Gursan O, Akar MS, Guvencer M, Havitcioglu H, et al. Anatomic footprint of the direct head of the rectus femoris origin: cadaveric study and clinical series of hips after arthroscopic anterior inferior iliac spine/subspine decompression. Arthroscopy : the journal of arthroscopic & related surgery : official publication of the Arthroscopy Association of North America and the International Arthroscopy Association. 2013;29(12):1932-40.

11. McCormick F, Alpaugh K, Nwachukwu BU, Yanke AB, Martin SD. Endoscopic repair of full-thickness abductor tendon tears: surgical technique and outcome at minimum of 1-year follow-up. Arthroscopy : the journal of arthroscopic & related surgery : official publication of the Arthroscopy Association of North America and the International Arthroscopy Association. 2013;29(12):1941-7.

12. Bogunovic L, Gottlieb M, Pashos G, Baca G, Clohisy JC. Why do hip arthroscopy procedures fail? Clinical orthopaedics and related research. 2013;471(8):2523-9.

13. Spencer-Gardner L, Eischen JJ, Levy BA, Sierra RJ, Engasser WM, Krych AJ. A comprehensive five-phase rehabilitation programme after hip arthroscopy for femoroacetabular impingement. Knee surgery, sports traumatology, arthroscopy : official journal of the ESSKA. 2014;22(4):848-59.

14. Ochs BC, Herzka A, Yaylali I. Intraoperative neurophysiological monitoring of somatosensory evoked potentials during hip arthroscopy surgery. The Neurodiagnostic journal. 2012;52(4):312-9.

15. Philippon MJ, Briggs KK, Carlisle JC, Patterson DC. Joint space predicts THA after hip arthroscopy in patients 50 years and older. Clinical orthopaedics and related research. 2013;471(8):2492-6.

16. Krych AJ, Baran S, Kuzma SA, Smith HM, Johnson RL, Levy BA. Utility of multimodal analgesia with fascia iliaca blockade for acute pain management following hip arthroscopy. Knee surgery, sports traumatology, arthroscopy : official journal of the ESSKA. 2014;22(4):843-7.

17. McCormick F, Nwachukwu BU, Alpaugh K, Martin SD. Predictors of hip arthroscopy outcomes for labral tears at minimum 2-year follow-up: the influence of age and arthritis. Arthroscopy : the journal of arthroscopic & related surgery : official publication of the Arthroscopy Association of North America and the International Arthroscopy Association. 2012;28(10):1359-64.

18. McCormick F, Slikker W, 3rd, Harris JD, Gupta AK, Abrams GD, Frank J, et al. Evidence of capsular defect following hip arthroscopy. Knee surgery, sports traumatology, arthroscopy : official journal of the ESSKA. 2014;22(4):902-5.

19. Boykin RE, Patterson D, Briggs KK, Dee A, Philippon MJ. Results of arthroscopic labral reconstruction of the hip in elite athletes. The American journal of sports medicine. 2013;41(10):2296-301.

20. Stake CE, Jackson TJ, Stone JC, Domb BG. Hip arthroscopy for labral tears in workers' compensation: a matched-pair controlled study. The American journal of sports medicine. 2013;41(10):2302-7.

21. YaDeau JT, Tedore T, Goytizolo EA, Kim DH, Green DS, Westrick A, et al. Lumbar plexus blockade reduces pain after hip arthroscopy: a prospective randomized controlled trial. Anesthesia and analgesia. 2012;115(4):968-72.

22. Cascio BM, King D, Yen YM. Psoas impingement causing labrum tear: a series from three tertiary hip arthroscopy centers. The Journal of the Louisiana State Medical Society : official organ of the Louisiana State Medical Society. 2013;165(2):88-93.

23. Freeman CR, Jones K, Byrd JW. Hip arthroscopy for Legg-Calve-Perthes disease: minimum 2-year follow-up. Arthroscopy : the journal of arthroscopic & related surgery : official publication of the Arthroscopy Association of North America and the International Arthroscopy Association. 2013;29(4):666-74.

24. Geyer MR, Philippon MJ, Fagrelius TS, Briggs KK. Acetabular labral reconstruction with an iliotibial band autograft: outcome and survivorship analysis at minimum 3-year follow-up. The American journal of sports medicine. 2013;41(8):1750-6.

25. McDonald JE, Herzog MM, Philippon MJ. Return to play after hip arthroscopy with microfracture in elite athletes. Arthroscopy : the journal of arthroscopic & related surgery : official publication of the Arthroscopy Association of North America and the International Arthroscopy Association. 2013;29(2):330-5.

26. Matsuda DK, Burchette RJ. Arthroscopic hip labral reconstruction with a gracilis autograft versus labral refixation: 2-year minimum outcomes. The American journal of sports medicine. 2013;41(5):980-7.

27. Matsuda DK, Calipusan CP. Adolescent femoroacetabular impingement from malunion of the anteroinferior iliac spine apophysis treated with arthroscopic spinoplasty. Orthopedics. 2012;35(3):e460-3.

28. Matsuda DK, Khatod M. Rapidly progressive osteoarthritis after arthroscopic labral repair in patients with hip dysplasia. Arthroscopy : the journal of arthroscopic & related surgery : official publication of the Arthroscopy Association of North America and the International Arthroscopy Association. 2012;28(11):1738-43.

29. Rupp R, Duggan B. Peripheral versus central compartment starting point in hip arthroscopy for femoroacetabular impingement. Orthopedics. 2012;35(2):e148-53.

30. Lansford T, Munns SW. Arthroscopic treatment of Pipkin type I femoral head fractures: a report of 2 cases. Journal of orthopaedic trauma. 2012;26(7):e94-6.

31. Kalore NV, Jiranek WA. Save the torn labrum in hips with borderline acetabular coverage. Clinical orthopaedics and related research. 2012;470(12):3406-13.

32. Krych AJ, Thompson M, Larson CM, Byrd JW, Kelly BT. Is posterior hip instability associated with cam and pincer deformity? Clinical orthopaedics and related research. 2012;470(12):3390-7.

33. Ward JP, Albert DB, Altman R, Goldstein RY, Cuff G, Youm T. Are femoral nerve blocks effective for early postoperative pain management after hip arthroscopy? Arthroscopy : the journal of arthroscopic & related surgery : official publication of the Arthroscopy Association of North America and the International Arthroscopy Association. 2012;28(8):1064-9.

34. Philippon MJ, Ejnisman L, Ellis HB, Briggs KK. Outcomes 2 to 5 years following hip arthroscopy for femoroacetabular impingement in the patient aged 11 to 16 years. Arthroscopy : the journal of arthroscopic & related surgery : official publication of the Arthroscopy Association of North America and the International Arthroscopy Association. 2012;28(9):1255-61.

35. Philippon MJ, Schroder ESBG, Briggs KK. Hip arthroscopy for femoroacetabular impingement in patients aged 50 years or older. Arthroscopy : the journal of arthroscopic & related surgery : official publication of the Arthroscopy Association of North America and the International Arthroscopy Association. 2012;28(1):59-65.

36. Mei-Dan O, McConkey MO, Brick M. Catastrophic failure of hip arthroscopy due to iatrogenic instability: can partial division of the ligamentum teres and iliofemoral ligament cause subluxation? Arthroscopy : the journal of arthroscopic & related surgery : official publication of the Arthroscopy Association of North America and the International Arthroscopy Association. 2012;28(3):440-5.

37. Bedi A, Zbeda RM, Bueno VF, Downie B, Dolan M, Kelly BT. The incidence of heterotopic ossification after hip arthroscopy. The American journal of sports medicine. 2012;40(4):854-63.

38. Meftah M, Rodriguez JA, Panagopoulos G, Alexiades MM. Long-term results of arthroscopic labral debridement: predictors of outcomes. Orthopedics. 2011;34(10):e588-92.

39. Alaia MJ, Zuskov A, Davidovitch RI. Contralateral deep venous thrombosis after hip arthroscopy. Orthopedics. 2011;34(10):e674-7.

40. Nwachukwu BU, McFeely ED, Nasreddine AY, Krcik JA, Frank J, Kocher MS. Complications of hip arthroscopy in children and adolescents. Journal of pediatric orthopedics. 2011;31(3):227-31.

41. Ayeni OR, Bedi A, Lorich DG, Kelly BT. Femoral neck fracture after arthroscopic management of femoroacetabular impingement: a case report. The Journal of bone and joint surgery American volume. 2011;93(9):e47.

42. Schroeder KM, Donnelly MJ, Anderson BM, Ford MP, Keene JS. The analgesic impact of preoperative lumbar plexus blocks for hip arthroscopy. A retrospective review. Hip international : the journal of clinical and experimental research on hip pathology and therapy. 2013;23(1):93-8.

43. Domb BG, Shindle MK, McArthur B, Voos JE, Magennis EM, Kelly BT. Iliopsoas impingement: a newly identified cause of labral pathology in the hip. HSS journal : the musculoskeletal journal of Hospital for Special Surgery. 2011;7(2):145-50.

44. McCarthy JC, Jarrett BT, Ojeifo O, Lee JA, Bragdon CR. What factors influence long-term survivorship after hip arthroscopy? Clinical orthopaedics and related research. 2011;469(2):362-71.

45. Larson CM, Giveans MR, Taylor M. Does arthroscopic FAI correction improve function with radiographic arthritis? Clinical orthopaedics and related research. 2011;469(6):1667-76.

46. Byrd JW, Jones KS. Arthroscopic management of femoroacetabular impingement: minimum 2-year follow-up. Arthroscopy : the journal of arthroscopic & related surgery : official publication of the Arthroscopy Association of North America and the International Arthroscopy Association. 2011;27(10):1379-88.

47. Badylak JS, Keene JS. Do iatrogenic punctures of the labrum affect the clinical results of hip arthroscopy? Arthroscopy : the journal of arthroscopic & related surgery : official publication of the Arthroscopy Association of North America and the International Arthroscopy Association. 2011;27(6):761-7.

48. Salvo JP, Troxell CR, Duggan DP. Incidence of venous thromboembolic disease following hip arthroscopy. Orthopedics. 2010;33(9):664.

49. Scher DL, Belmont PJ, Jr., Owens BD. Case report: Osteonecrosis of the femoral head after hip arthroscopy. Clinical orthopaedics and related research. 2010;468(11):3121-5.

50. Verma M, Sekiya JK. Intrathoracic fluid extravasation after hip arthroscopy. Arthroscopy : the journal of arthroscopic & related surgery : official publication of the Arthroscopy Association of North America and the International Arthroscopy Association. 2010;26(9 Suppl):S90-4.

51. Ladner B, Nester K, Cascio B. Abdominal fluid extravasation during hip arthroscopy. Arthroscopy : the journal of arthroscopic & related surgery : official publication of the Arthroscopy Association of North America and the International Arthroscopy Association. 2010;26(1):131-5.

52. Fowler J, Owens BD. Abdominal compartment syndrome after hip arthroscopy. Arthroscopy : the journal of arthroscopic & related surgery : official publication of the Arthroscopy Association of North America and the International Arthroscopy Association. 2010;26(1):128-30.

53. Philippon MJ, Weiss DR, Kuppersmith DA, Briggs KK, Hay CJ. Arthroscopic labral repair and treatment of femoroacetabular impingement in professional hockey players. The American journal of sports medicine. 2010;38(1):99-104.

54. Philippon MJ, Ferro FP, Nepple JJ. Hip capsulolabral spacer placement for the treatment of severe capsulolabral adhesions after hip arthroscopy. Arthroscopy techniques. 2014;3(2):e289-92.

55. Sekiya JK, Martin RL, Lesniak BP. Arthroscopic repair of delaminated acetabular articular cartilage in femoroacetabular impingement. Orthopedics. 2009;32(9).

56. Bushnell BD, Dahners LE. Fatal pulmonary embolism in a polytraumatized patient following hip arthroscopy. Orthopedics. 2009;32(1):56.

57. Hetsroni I, Dela Torre K, Duke G, Lyman S, Kelly BT. Sex differences of hip morphology in young adults with hip pain and labral tears. Arthroscopy : the journal of arthroscopic & related surgery : official publication of the Arthroscopy Association of North America and the International Arthroscopy Association. 2013;29(1):54-63.

58. Hetsroni I, Larson CM, Dela Torre K, Zbeda RM, Magennis E, Kelly BT. Anterior inferior iliac spine deformity as an extra-articular source for hip impingement: a series of 10 patients treated with arthroscopic decompression. Arthroscopy : the journal of arthroscopic & related surgery : official publication of the Arthroscopy Association of North America and the International Arthroscopy Association. 2012;28(11):1644-53.

59. Feeley BT, Kelly BT. Arthroscopic management of an intraarticular osteochondroma of the hip. Orthopedic reviews. 2009;1(1):e2.

60. Ranawat AS, McClincy M, Sekiya JK. Anterior dislocation of the hip after arthroscopy in a patient with capsular laxity of the hip. A case report. The Journal of bone and joint surgery American volume. 2009;91(1):192-7.

61. Parvizi J, Bican O, Bender B, Mortazavi SM, Purtill JJ, Erickson J, et al. Arthroscopy for labral tears in patients with developmental dysplasia of the hip: a cautionary note. The Journal of arthroplasty. 2009;24(6 Suppl):110-3.

62. Nepple JJ, Zebala LP, Clohisy JC. Labral disease associated with femoroacetabular impingement: do we need to correct the structural deformity? The Journal of arthroplasty. 2009;24(6 Suppl):114-9.

63. Matsuda DK. Acute iatrogenic dislocation following hip impingement arthroscopic surgery. Arthroscopy : the journal of arthroscopic & related surgery : official publication of the Arthroscopy Association of North America and the International Arthroscopy Association. 2009;25(4):400-4.

64. Sharma A, Sachdev H, Gomillion M. Abdominal compartment syndrome during hip arthroscopy. Anaesthesia. 2009;64(5):567-9.

65. Martin HD, Palmer IJ, Champlin K, Kaiser B, Kelly B, Leunig M. Physiological changes as a result of hip arthroscopy performed with traction. Arthroscopy : the journal of arthroscopic & related surgery : official publication of the Arthroscopy Association of North America and the International Arthroscopy Association. 2012;28(10):1365-72.

66. Anderson SA, Keene JS. Results of arthroscopic iliopsoas tendon release in competitive and recreational athletes. The American journal of sports medicine. 2008;36(12):2363-71.

67. McCarthy JJ, MacEwen GD. Hip arthroscopy for the treatment of children with hip dysplasia: a preliminary report. Orthopedics. 2007;30(4):262-4.

68. Philippon M, Schenker M, Briggs K, Kuppersmith D. Femoroacetabular impingement in 45 professional athletes: associated pathologies and return to sport following arthroscopic decompression. Knee surgery, sports traumatology, arthroscopy : official journal of the ESSKA. 2007;15(7):908-14.

69. Kocher MS, Kim YJ, Millis MB, Mandiga R, Siparsky P, Micheli LJ, et al. Hip arthroscopy in children and adolescents. Journal of pediatric orthopedics. 2005;25(5):680-6.

70. Byrd JW, Jones KS. Hip arthroscopy in the presence of dysplasia. Arthroscopy : the journal of arthroscopic & related surgery : official publication of the Arthroscopy Association of North America and the International Arthroscopy Association. 2003;19(10):1055-60.

71. Svoboda SJ, Williams DM, Murphy KP. Hip arthroscopy for osteochondral loose body removal after a posterior hip dislocation. Arthroscopy : the journal of arthroscopic & related surgery : official publication of the Arthroscopy Association of North America and the International Arthroscopy Association. 2003;19(7):777-81.

72. Mineo RC, Gittins ME. Arthroscopic removal of a bullet embedded in the acetabulum. Arthroscopy : the journal of arthroscopic & related surgery : official publication of the Arthroscopy Association of North America and the International Arthroscopy Association. 2003;19(9):E121-24.

73. Meyer NJ, Thiel B, Ninomiya JT. Retrieval of an intact, intraarticular bullet by hip arthroscopy using the lateral approach. Journal of orthopaedic trauma. 2002;16(1):51-3.

74. Byrd JW, Jones KS. Osteoarthritis caused by an inverted acetabular labrum: radiographic diagnosis and arthroscopic treatment. Arthroscopy : the journal of arthroscopic & related surgery : official publication of the Arthroscopy Association of North America and the International Arthroscopy Association. 2002;18(7):741-7.

75. Sampson TG. Complications of hip arthroscopy. Clinics in sports medicine. 2001;20(4):831-5.

76. Bartlett CS, DiFelice GS, Buly RL, Quinn TJ, Green DS, Helfet DL. Cardiac arrest as a result of intraabdominal extravasation of fluid during arthroscopic removal of a loose body from the hip joint of a patient with an acetabular fracture. Journal of orthopaedic trauma. 1998;12(4):294-9.

77. Ilizaliturri VM, Jr., Chaidez C, Villegas P, Briseno A, Camacho-Galindo J. Prospective randomized study of 2 different techniques for endoscopic iliopsoas tendon release in the treatment of internal snapping hip syndrome. Arthroscopy : the journal of arthroscopic & related surgery : official publication of the Arthroscopy Association of North America and the International Arthroscopy Association. 2009;25(2):159-63.

78. Wilkin G, March G, Beaule PE. Arthroscopic acetabular labral debridement in patients forty-five years of age or older has minimal benefit for pain and function. The Journal of bone and joint surgery American volume. 2014;96(2):113-8.

79. Chan K, Farrokhyar F, Burrow S, Kowalczuk M, Bhandari M, Ayeni OR. Complications following hip arthroscopy: a retrospective review of the McMaster experience (2009-2012). Canadian journal of surgery Journal canadien de chirurgie. 2013;56(6):422-6.

80. Gaudelli C, Mohtadi N. Pulmonary embolism after hip arthroscopy. Knee surgery, sports traumatology, arthroscopy : official journal of the ESSKA. 2011;19(7):1224-5.

81. Trompeter A, Colegate-Stone T, Khakha R, Hull J. Hip arthroscopy for femoroacetabular impingement: results of 118 consecutive cases in a district general hospital. Hip international : the journal of clinical and experimental research on hip pathology and therapy. 2013;23(4):400-5.

82. Karthikeyan S, Roberts S, Griffin D. Microfracture for acetabular chondral defects in patients with femoroacetabular impingement: results at second-look arthroscopic surgery. The American journal of sports medicine. 2012;40(12):2725-30.

83. Gaymer CE, Achten J, Auckett R, Cooper L, Griffin D. Fluoroscopic radiation exposure during hip arthroscopy. Arthroscopy : the journal of arthroscopic & related surgery : official publication of the Arthroscopy Association of North America and the International Arthroscopy Association. 2013;29(5):870-3.

84. Aprato A, Jayasekera N, Villar R. Timing in hip arthroscopy: does surgical timing change clinical results? International orthopaedics. 2012;36(11):2231-4.

85. Aprato A, Jayasekera N, Villar RN. Does the modified Harris hip score reflect patient satisfaction after hip arthroscopy? The American journal of sports medicine. 2012;40(11):2557-60.

86. Jayasekera N, Aprato A, Villar RN. Are crutches required after hip arthroscopy? A case-control study. Hip international : the journal of clinical and experimental research on hip pathology and therapy. 2013;23(3):269-73.

87. Malviya A, Stafford GH, Villar RN. Is hip arthroscopy for femoroacetabular impingement only for athletes? British journal of sports medicine. 2012;46(14):1016-8.

88. Vendittoli PA, Young DA, Stitson DJ, Wolfe R, Del Buono A, Maffulli N. Acetabular rim lesions: arthroscopic assessment and clinical relevance. International orthopaedics. 2012;36(11):2235-41.

89. Cooper AP, Basheer SZ, Maheshwari R, Regan L, Madan SS. Outcomes of hip arthroscopy. A prospective analysis and comparison between patients under 25 and over 25 years of age. British journal of sports medicine. 2013;47(4):234-8.

90. Schilders E, Dimitrakopoulou A, Bismil Q, Marchant P, Cooke C. Arthroscopic treatment of labral tears in femoroacetabular impingement: a comparative study of refixation and resection with a minimum two-year follow-up. The Journal of bone and joint surgery British volume. 2011;93(8):1027-32.

91. Javed A, O'Donnell JM. Arthroscopic femoral osteochondroplasty for cam femoroacetabular impingement in patients over 60 years of age. The Journal of bone and joint surgery British volume. 2011;93(3):326-31.

92. Stafford GH, Malviya A, Villar RN. Fluid extravasation during hip arthroscopy. Hip international : the journal of clinical and experimental research on hip pathology and therapy. 2011;21(6):740-3.

93. Field RE, Rajakulendran K, Strambi F. Arthroscopic grafting of chondral defects and subchondral cysts of the acetabulum. Hip international : the journal of clinical and experimental research on hip pathology and therapy. 2011;21(4):479-86.

94. Konan S, Rhee SJ, Haddad FS. Hip arthroscopy: analysis of a single surgeon's learning experience. The Journal of bone and joint surgery American volume. 2011;93 Suppl 2:52-6.

95. Tzaveas AP, Villar RN. Arthroscopic repair of acetabular chondral delamination with fibrin adhesive. Hip international : the journal of clinical and experimental research on hip pathology and therapy. 2010;20(1):115-9.

96. Clarke MT, Arora A, Villar RN. Hip arthroscopy: complications in 1054 cases. Clinical orthopaedics and related research. 2003(406):84-8.

97. Eberhardt O, Fernandez FF, Wirth T. Arthroscopic reduction of the dislocated hip in infants. The Journal of bone and joint surgery British volume. 2012;94(6):842-7.

98. Anders HJ. Pigmented villonodular synovitis of the hip in systemic lupus erythematosus: a case report. Journal of medical case reports. 2011;5:443.

99. Said HG, Steimer O, Kohn D, Dienst M. Vascular obstruction at the level of the ankle joint as a complication of hip arthroscopy. Arthroscopy : the journal of arthroscopic & related surgery : official publication of the Arthroscopy Association of North America and the International Arthroscopy Association. 2011;27(11):1594-6.

100. Streich NA, Gotterbarm T, Barie A, Schmitt H. Prognostic value of chondral defects on the outcome after arthroscopic treatment of acetabular labral tears. Knee surgery, sports traumatology, arthroscopy : official journal of the ESSKA. 2009;17(10):1257-63.

101. Benali Y, Katthagen BD. Hip subluxation as a complication of arthroscopic debridement. Arthroscopy : the journal of arthroscopic & related surgery : official publication of the Arthroscopy Association of North America and the International Arthroscopy Association. 2009;25(4):405-7.

102. Jerosch J, Schunck J. Arthroscopic treatment of lateral epicondylitis: indication, technique and early results. Knee surgery, sports traumatology, arthroscopy : official journal of the ESSKA. 2006;14(4):379-82.

103. Dienst M, Godde S, Seil R, Hammer D, Kohn D. Hip arthroscopy without traction: In vivo anatomy of the peripheral hip joint cavity. Arthroscopy : the journal of arthroscopic & related surgery : official publication of the Arthroscopy Association of North America and the International Arthroscopy Association. 2001;17(9):924-31.

104. Zingg PO, Ulbrich EJ, Buehler TC, Kalberer F, Poutawera VR, Dora C. Surgical hip dislocation versus hip arthroscopy for femoroacetabular impingement: clinical and morphological short-term results. Archives of orthopaedic and trauma surgery. 2013;133(1):69-79.

105. Leunig M, Mast NH, Impellizerri FM, Ganz R, Panaro C. Arthroscopic appearance and treatment of impingement cysts at femoral head-neck junction. Arthroscopy : the journal of arthroscopic & related surgery : official publication of the Arthroscopy Association of North America and the International Arthroscopy Association. 2012;28(1):66-73.

106. Buchler L, Neumann M, Schwab JM, Iselin L, Tannast M, Beck M. Arthroscopic versus open cam resection in the treatment of femoroacetabular impingement. Arthroscopy : the journal of arthroscopic & related surgery : official publication of the Arthroscopy Association of North America and the International Arthroscopy Association. 2013;29(4):653-60.

107. Stahelin L, Stahelin T, Jolles BM, Herzog RF. Arthroscopic offset restoration in femoroacetabular cam impingement: accuracy and early clinical outcome. Arthroscopy : the journal of arthroscopic & related surgery : official publication of the Arthroscopy Association of North America and the International Arthroscopy Association. 2008;24(1):51-7 e1.

108. Horisberger M, Brunner A, Herzog RF. Arthroscopic treatment of femoral acetabular impingement in patients with preoperative generalized degenerative changes. Arthroscopy : the journal of arthroscopic & related surgery : official publication of the Arthroscopy Association of North America and the International Arthroscopy Association. 2010;26(5):623-9.

109. Haupt U, Volkle D, Waldherr C, Beck M. Intra- and retroperitoneal irrigation liquid after arthroscopy of the hip joint. Arthroscopy : the journal of arthroscopic & related surgery : official publication of the Arthroscopy Association of North America and the International Arthroscopy Association. 2008;24(8):966-8.

110. Funke EL, Munzinger U. Complications in hip arthroscopy. Arthroscopy : the journal of arthroscopic & related surgery : official publication of the Arthroscopy Association of North America and the International Arthroscopy Association. 1996;12(2):156-9.

111. Pailhe R, Chiron P, Reina N, Cavaignac E, Lafontan V, Laffosse JM. Pudendal nerve neuralgia after hip arthroscopy: retrospective study and literature review. Orthopaedics & traumatology, surgery & research : OTSR. 2013;99(7):785-90.

112. Flecher X, Dumas J, Argenson JN. Is a hip distractor useful in the arthroscopic treatment of femoroacetabular impingement? Orthopaedics & traumatology, surgery & research : OTSR. 2011;97(4):381-8.

113. Gedouin JE, May O, Bonin N, Nogier A, Boyer T, Sadri H, et al. Assessment of arthroscopic management of femoroacetabular impingement. A prospective multicenter study. Orthopaedics & traumatology, surgery & research : OTSR. 2010;96(8 Suppl):S59-67.

114. Boyer T, Dorfmann H. Arthroscopy in primary synovial chondromatosis of the hip: description and outcome of treatment. The Journal of bone and joint surgery British volume. 2008;90(3):314-8.

115. Zini R, Munegato D, De Benedetto M, Carraro A, Bigoni M. Endoscopic iliotibial band release in snapping hip. Hip international : the journal of clinical and experimental research on hip pathology and therapy. 2013;23(2):225-32.

116. Bruno M, Longhino V, Sansone V. A catastrophic complication of hip arthroscopy. Arthroscopy : the journal of arthroscopic & related surgery : official publication of the Arthroscopy Association of North America and the International Arthroscopy Association. 2011;27(8):1150-2.

117. Randelli F, Randelli P, Banci L, Arrigoni P. Intra-articular loose body removal during hip arthroscopy. Orthopedics. 2010;33(7):476.

118. Randelli F, Pierannunzii L, Banci L, Ragone V, Aliprandi A, Buly R. Heterotopic ossifications after arthroscopic management of femoroacetabular impingement: the role of NSAID prophylaxis. Journal of orthopaedics and traumatology : official journal of the Italian Society of Orthopaedics and Traumatology. 2010;11(4):245-50.

119. Verhelst L, De Schepper J, Sergeant G, Liekens K, Delport H. Variations in serum electrolyte concentrations and renal function after therapeutic hip arthroscopy: a pilot study. Arthroscopy : the journal of arthroscopic & related surgery : official publication of the Arthroscopy Association of North America and the International Arthroscopy Association. 2009;25(4):377-81.

120. Alvarez MS, Moneo PR, Palacios JA. Arthroscopic extirpation of an osteoid osteoma of the acetabulum. Arthroscopy : the journal of arthroscopic & related surgery : official publication of the Arthroscopy Association of North America and the International Arthroscopy Association. 2001;17(7):768-71.

121. Sener N, Gogus A, Akman S, Hamzaoglu A. Avascular necrosis of the femoral head after hip arthroscopy. Hip international : the journal of clinical and experimental research on hip pathology and therapy. 2011;21(5):623-6.

122. Sozen YV, Polat G, Kadioglu B, Dikici F, Ozkan K, Unay K. Arthroscopic bullet extraction from the hip in the lateral decubitus position. Hip international : the journal of clinical and experimental research on hip pathology and therapy. 2010;20(2):265-8.

123. Kim SJ, Choi NH, Kim HJ. Operative hip arthroscopy. Clinical orthopaedics and related research. 1998(353):156-65.

124. Wang WG, Yue DB, Zhang NF, Hong W, Li ZR. Clinical diagnosis and arthroscopic treatment of acetabular labral tears. Orthopaedic surgery. 2011;3(1):28-34.

125. Yamamoto Y, Ide T, Ono T, Hamada Y. Usefulness of arthroscopic surgery in hip trauma cases. Arthroscopy : the journal of arthroscopic & related surgery : official publication of the Arthroscopy Association of North America and the International Arthroscopy Association. 2003;19(3):269-73.

126. Lo YP, Chan YS, Lien LC, Lee MS, Hsu KY, Shih CH. Complications of hip arthroscopy: analysis of seventy three cases. Chang Gung medical journal. 2006;29(1):86-92.

127. Gupta RK, Aggarwal V. Late arthroscopic retrieval of a bullet from hip joint. Indian journal of orthopaedics. 2009;43(4):416-9.

128. Amenabar T, O'Donnell J. Return to sport in Australian football league footballers after hip arthroscopy and midterm outcome. Arthroscopy : the journal of arthroscopic & related surgery : official publication of the Arthroscopy Association of North America and the International Arthroscopy Association. 2013;29(7):1188-94.

129. Haviv B, O'Donnell J. Arthroscopic treatment for symptomatic bilateral cam-type femoroacetabular impingement. Orthopedics. 2010;33(12):874.

130. Singh PJ, Constable L, O'Donnell J. Arthroscopic excision of a giant-cell tumour of the ligamentum teres. The Journal of bone and joint surgery British volume. 2009;91(6):809-11.

131. Walton NP, Jahromi I, Lewis PL. Chondral degeneration and therapeutic hip arthroscopy. International orthopaedics. 2004;28(6):354-6.

132. Contreras ME, Dani WS, Endges WK, De Araujo LC, Berral FJ. Arthroscopic treatment of the snapping iliopsoas tendon through the central compartment of the hip: a pilot study. The Journal of bone and joint surgery British volume. 2010;92(6):777-80.

133. Souza BG, Dani WS, Honda EK, Ricioli W, Jr., Guimaraes RP, Ono NK, et al. Do complications in hip arthroscopy change with experience? Arthroscopy : the journal of arthroscopic & related surgery : official publication of the Arthroscopy Association of North America and the International Arthroscopy Association. 2010;26(8):1053-7.

134. Parodi D, Tobar C, Valderrama J, Sauthier E, Besomi J, Lopez J, et al. Hip arthroscopy and hypothermia. Arthroscopy : the journal of arthroscopic & related surgery : official publication of the Arthroscopy Association of North America and the International Arthroscopy Association. 2012;28(7):924-8.
